# Palliative care for patients with heart failure: facilitators and barriers - a cross sectional survey of German health care professionals

**DOI:** 10.1186/s12913-016-1609-x

**Published:** 2016-08-08

**Authors:** Jeanette Ziehm, Erik Farin, Jonas Schäfer, Kathrin Woitha, Gerhild Becker, Stefan Köberich

**Affiliations:** 1 Section of Health Care Research and Rehabilitation Research, Medical Center – University of Freiburg, Faculty of Medicine, University of Freiburg, Engelbergerstr. 21, 79106 Freiburg, Germany; 2 Department of Palliative Care, Medical Center – University of Freiburg, Faculty of Medicine, University of Freiburg, Robert-Koch-Str. 3, 79106 Freiburg, Germany; 3 Department of Thoracic Surgery, Medical Center – University of Freiburg, Faculty of Medicine, University of Freiburg, Hugstetter Str. 55, 79106 Freiburg, Germany; 4 Pflegedirektion, Heart Center – University of Freiburg, Hugstetter Str. 55, 79106 Freiburg, Germany

**Keywords:** Palliative care, Heart failure, Healthcare professionals, Attitudes, Experiences, Survey, Germany

## Abstract

**Background:**

Compared to patients with cancer, heart failure patients are seldom candidates for palliative care. Numerous studies have investigated reasons why heart failure patients do not receive palliative care; however, none of these studies have ever evaluated the situation in the German health care setting. This study aims to identify German healthcare providers’ (HCP) perception of barriers and facilitators to palliative care of patients with chronic heart failure.

**Methods:**

We conducted an online-survey with 315 nurses and physicians of different medical disciplines.

**Results:**

Even though heart failure patients’ need of palliative care and its advantages has been recognized, HCP see potential for development and improvement. A lack of knowledge about the content and measures of palliative care, poor communication and unclear responsibilities between medical disciplines, difficulties to determine the right time to initiate palliative care, and the feeling not to be prepared to discuss end-of-life issues with the patient has been identified as barriers. Further, HCP believe that patients and relatives do not possess adequate knowledge about the disease and its progression and are therefore unprepared in asking questions regarding palliative care. They rather tend to demand everything possible to be done in order prolong life, and are reluctant to accept that life is limited. Overall, HCP perceive that dying is a taboo subject within our society placing palliative care on the same level as assisted dying. In addition, results indicate that HCP have an inappropriate notion of ideal medicine fearing to lose patient and are worried about the appropriateness of PC remuneration.

**Conclusions:**

In order to overcome the described barriers, HCP, patients, and relatives need to be educated in palliative care. Information and education encompassing the aim, content and measures of palliative care needs to be provided for all parties in order to optimize patient care, to foster communication between healthcare professionals, patients, and relatives, and to overcome perceived barriers.

**Trial registration:**

DRKS00007119

**Electronic supplementary material:**

The online version of this article (doi:10.1186/s12913-016-1609-x) contains supplementary material, which is available to authorized users.

## Background

In Germany, the age- and sex-standardized prevalence rates of CHF ranged from 1.7 to 1.9 between 2004-2006 [1]. CHF was the fourth leading cause for death in 2014 [2]. Due to an ageing society, it is expected that the total number of people with heart failure will rise in the next decades.

Patients with chronic heart failure (CHF) frequently suffer from symptoms like dyspnea [3], fatigue [4], cognitive impairment [5], and pain [6] which lead to a reduction of physical functioning, to a restriction in performing task of everyday life [7, 8], and in consequence to the need of help from others [9, 10]. Furthermore, CHF affects the lives of CHF patients’ caregivers. They suffer from e.g. social isolation [11], anxiety [12], sleep deprivation [13], and depression [14–16].

Even though symptom burden of patients with CHF is as high as for patients with malignant diseases [17–19], and the prevalence of CHF is rising, referral rates of CHF patients to palliative care (PC) fall short of referral rates of tumor patients [20–22]. There is also evidence that patients with CHF and their caregivers benefit from PC. Palliative care in terms of early identification, assessment and treatment of pain and other physical, psychosocial and spiritual problems [23] reduces CHF patients’ symptom burden and improves quality of life. In addition, the usage of opioids is decreased due to improved pain management [24]. Patients as well as caregivers are more satisfied with health care providers’ (HCP) care, both experiencing the care as more holistic and consider PC as a helpful support for effective coping [13, 24–29].

Reasons for non-referral to PC of patients with CHF have been investigated in numerous studies all over the world. A lack of continuity and coordination of CHF patients’ care, insufficient communication between HCPs [30, 31], difficulties in determining when to commence PC [31, 32], fear of HCP due to possible deprivation of patient’s hope when mentioning PC [31–33], the disagreement of HCP regarding responsibilities for PC [31, 34] as well as HCPs’ lack of knowledge about PC [32, 33] have been identified as barriers.

According to the World Health Organization, Germany is a country in which PC services are at a stage of advanced integration into mainstream service provision. A wide range of PC services and provision already exist, encompassing a broad awareness of PC on the part of health professionals, local communities and society in general [35]. However, there is also evidence that patients with CHF receive less PC than patients with malignant diseases. For example, in 2014 only 3.0 % of all patients treated by hospice or ambulatory PC teams had a cardiovascular disease as main diagnosis. In contrast 80.4 % of the patients had a main diagnosis of a malignant disease [36]. Reasons for differences in PC treatment between patients with oncological and non-oncological diseases have not yet been investigated in the German healthcare system. Therefore, the aim of this study was to investigate HCPs’ perceived barriers and facilitators of PC of patients with CHF.

## Methods

### Design

For the purpose of study we used a cross-sectional, survey design.

### Instrument

The questionnaire (Additional file 1) was developed based on a previous qualitative study in which 23 interviews with German HCP (physicians, nurses) were conducted [37] and analyzed using Qualitative Content Analysis as described by Mayring [38]. Aim of this study was to evaluate participants’ attitude towards and experience with PC of CHF patients as well as perceived barriers and facilitators to PC.

Analysis of the interviews showed, that a lack of knowledge of palliative care, shortcomings in communication and cooperation between health care professionals, the inability of cardiologist to accept medical limits and death as an entity of CHF, and the difficulty to determine the right time to initiate PC in CHF patients were identified as barriers to PC. Participants of this study part expressed their hope that better communication and cooperation between the medical disciplines as well as education about PC and CHF for physicians, nurses, patients and for the public would foster PC for patients with CHF.

Participants’ statements were analyzed and grouped into categories and subcategories. The categories and the inherent statements were then transferred as items into the questionnaire. After formulating the items, a first version of the questionnaire with 72 items was piloted with three nurses and two physicians, and then modified according to the given feedback. A further section was added to collect socio-demographic variables of the participants.

Overall, the final questionnaire consists of 67 items, divided into five sections (Table 1). Items could be answered using a 5-point Likert scale (1 = “fully agree” to 5 = “fully disagree”). After each section comment fields for additional remarks were included. Two additional questions were added to the questionnaire. The participants were asked what their opinions were on the usefulness of PC in treating CHF patients and if the distinction between general PC and specialized PC (as it exists in Germany) is known by German HCPs. In Germany, general PC is conducted by the primary health care providers (e.g. general practitioner, outpatient nursing care services) aiming to maintain patients’ quality of life and self-determination and to facilitate a dignified life until death. If the therapeutic possibilities are not sufficient to reach this aim specialized palliative care teams should be involved [39].

**Table 1:** Thematic content of questionnaire

Themes	Number of Items	Answer categories
Aims of PC	9	5-point Likert-Scale^a^
Organizational conditions of PC	16	5-point Likert-Scale^a^
Barriers to PC	18	5-point Likert-Scale^a^
Attitudes towards PC	14	5-point Likert-Scale^a^
Time to start PC	10	5-point Likert-Scale^a^
Is PC useful for CHF patients	1	Yes/No/No opinion
Is difference between general/special PC known?	1	Yes/No

^a^ 1 = fully agree to 5 = fully disagree

Abbr.: PC = palliative care

The questionnaire was designed as an online-questionnaire. The online platform for the questionnaire was provided by Unipark (www.unipark.de).

### Sample

A convenience sample of HCP who are involved in heart failure care answered the questionnaire. The sample was recruited using different approaches. A call for participation was placed in different professional journals and newsletters, via social media (Twitter), as well as on the homepage of the researcher’s institute. In addition emails were sent to research collaborations, to general practitioners (GPs), resident cardiologists and resident internists, to outpatient nursing services, to professional associations, to medical and nursing directors of German heart centers and to persons from the personal and professional network of the researchers. Those who were emailed were also asked to forward the call to friends and colleagues.

The questionnaire was online during a twelve week period from July to September 2015.

### Statistics

The data were exported from unipark.de to SPSS for Windows Version 22. Only completed data sets were included in the analysis.

We dichotomized the possible answers for reading purposes. “Fully agree” and “agree” were recoded as “Agree” and “Neither nor”, “disagree” and “fully disagree” were recoded as “Disagree”. Rates of agreement are displayed in numbers and percentages.

Thematic analysis was applied to the qualitative written responses.

## Results

### Participants

The online questionnaire was called up 616 times. In 315 cases (51.1 %) none of the questions were answered. The questionnaire was partly completed by 126 participants (20.5 %) and fully completed by 175 participants (28.4 %).

Socio-demographic and professional data of the participants are displayed in Table 2. Overall, participants were predominantly male (n = 91; 52.0 %), physicians (n = 95; 54.3 %) and had an average age of 43.8 years (SD: 15.1).

**Table 2:** Sociodemographic and professional variables of participants

	N(%)
Age	43.8 (±15.1)
Gender	
Male	91 (52.0)
Female	76 (43.4)
Occupation	
Physician	95 (54.3)
General practitioner	46 (26.3)
Resident cardiologist	13 (7.4)
Hospital cardiologist	18 (10.3)
Others	18 (10.3)
Years of professional experience	20.0 (± 10.7)
Nurse	71 (40.6)
Nurse in hospital	50 (28.6)
Community nurse	19 (10.9)
Years of professional experience	18.5 (±11.7)

### Usefulness of palliative care of patients with chronic heart failure

The participants of this study were in favor of PC for patients with CHF. Most (n = 137; 78.3 %) thought that PC population, three (1.7%) did not share this point of view, and five (2.9 %) had no opinion regarding PC of CHF patients.

### Aims of palliative care

Participants’ evaluations regarding aims of PC are displayed in Table 3. They agreed that the aim of PC is to maintain the best possible quality of life and to provide a dignified death. Supporting the patient, relieving physical symptoms, and discomfort were seen as measures of PC.

**Table 3:** Aims of palliative care

Within palliative care…	Agreement
…communication with and involvement of the relatives plays an important role.	166 (94.9 %)
…an incurable patient will be cared for.	165 (94.3 %)
…the aim is to achieve/maintain best possible quality of life.	162 (92.6 %)
…the aim is a dignified death.	162 (92.6 %)
…psychological support is provided.	160 (91.4 %)
…one tries to avoid unnecessary therapy.	157 (89.7 %)
…extent and intensity of technical and life-sustaining measures well be deliberated and discussed with the patient.	156 (89.1 %)
…physical symptoms and discomfort as well as their relief are paramount.	153 (87.4 %)
…spiritual support will be provided.	145 (82.9 %)

Furthermore, interprofessional communication and communication with the patients and their relatives was seen as important for PC. Participants agreed that type and scope of life-sustaining measures should be discussed with the patient.

Additional comments by the participants suggest that the aim of PC should be to care for the patient at home (“Safeguard in the home setting”; #181) and emphasize that PC should be patient-centered (“[…] wishes and will of the patients have the highest priority”; #231). Participants’ statements reflect also the opinion that PC should always include all people affected by the disease of the patient, i.e. relatives and HCPs, which was reflected by one participant’s statement: “[Within palliative care] all significant persons involved with the patient should be included […]”; (#457). One participant stated that it is of great importance that the meaning and the aim of PC be discussed with the attending physician (#299). Another participant pointed out that PC should be paid off for those who are responsible for PC (#548).

### Organizational conditions of palliative care

Participants’ opinions regarding optimal organizational conditions for PC are displayed in Table 4. Participants believed that cooperation of all HCP and medical disciplines are essential for PC of patients with CHF, as well as interdisciplinary collaboration to create and maintain clarity. This was emphasized in various statements made by the participants. They wrote that “all physicians should work together and should exchange information about the patient in order to provide the best care in all stages of life and death” (#95) and that “therapy should be in consensus among all disciplines involved” (#52). One participant also stated that areas of responsibilities need to be clearly defined.

**Table 4:** Organizational conditions of palliative care

In favor of palliative care for patients with chronic heart failure….	Agreement
…cardiologist, general practitioner, palliative care practitioner and internist as well as nurses should work cooperatively together (meetings, case conferences, etc.).	161 (92.0 %)
…clear arrangements between all professionals/disciplines should be made.	161 (92.0 %)
…further education in the area of palliative care should be offered to all professions.	160 (91.4 %)
…collective interdisciplinary education should be offered to all physicians involved in caring for the patient.	157 (89.7 %)
…palliative care should be established within the institution (hospitals/long-term care facilities).	146 (83.4 %)
…palliative care practitioner should be available for consultation.	143 (81.7 %)
…palliative care should be initiated by the attending general practitioner.	133 (76.0 %)
…palliative care should be initiated by the attending cardiologist.	130 (74.3 %)
…palliative care should be initiated by a nurse.	128 (73.1 %)
…palliative care should be initiated by the attending internist.	118 (67.4 %)
..therapy should mainly be carried out by the palliative care practitioner.	90 (51.4 %)
..therapy should mainly be carried out by the attending general practitioner.	80 (45.7 %)
…palliative care should be initiated by the attending palliative care practitioner.	61 (34.9 %)
..therapy should mainly be carried out by the attending cardiologist.	48 (27.4 %)
..therapy should mainly be carry out by the attending internist.	46 (26.3 %)
…palliative care practitioner should have only an advisory role.	37 (21.1 %)

To achieve this aim, education for all HCP and collective education throughout all medical disciplines should be offered. Most participants agreed that PC should be established in acute (e.g., hospital) and long term care settings.

There was disagreement about who should be primarily responsible for initiation and conduct of PC for CHF patients. However, not all participants shared the opinion that responsibility for initiating PC needs to be clearly defined, which was reflected in the statement: “it is irrelevant [who] initiates PC treatment, rather, that someone carries it through” (#591).

There was also disagreement about who should primarily carry out the therapy. Participants’ comments which underline these findings are: “palliative care is the responsibility of the general practitioner” (#424), and “therapy should be carried out […] by a colleague with the closest working relationship to the patient. If there are any problems, the others should assist in an advisory or supportive capacity” (#52), or “treatment of the patient should be carried out in an interdisciplinary manner” (#249).

### Barriers to palliative care

As displayed in Table 5, HCP perceived that the lack of information provided to patients and relatives regarding the progression and severity of the patient’s disease (CHF) as being one of the main barriers. Consequently, the disease’s creeping course seems distant to patients, hence patients do not have the feeling to be in a palliative situation.

**Table 5:** Barriers to palliative care

Palliative care of patients with chronic heart failure is often hampered because…	Agreement
…patients and relatives are not sufficiently informed about the severity and the prognosis of CHF.	142 (81.1 %)
…physicians and nurses have an information deficit about content and possibilities of palliative care.	141 (80.6 %)
…it is easier to continue with an existing therapy than to discuss a change of the therapy’s aim with the patient.	132 (75.4 %)
…patients have a degree of reluctance in accepting that life is limited.	130 (74.3 %)
…in our society dying is a taboo subject.	128 (73.1 %)
…there are different attitudes between the different medical disciplines (cardiology, general medicine, internal medicine, palliative care medicine) regarding therapy of patients with heart failure.	127 (72.6 %)
…relatives want everything possible to be done.	125 (71.4 %)
..the creeping course of the disease does not look threatening.	124 (70.9 %)
…palliative care medicine mainly focuses on oncological patients.	115 (65.7 %)
..there are different attitudes between the different medical professions (nurses, physicians) regarding therapy of patients with heart failure.	112 (64.0 %)
…physicians/nurses do not have a palliative care contact person when needed.	111 (63.4 %)
…patients put palliative care medicine on the same level as euthanasia.	103 (58.9 %)
…patients want everything possible to be done.	101 (57.7 %)
… a lot of physicians perceive palliative care as a defeat.	86 (49.1 %)
…no palliative care approach exist for patients with heart failure.	76 (43.4 %)
…the medical team is not conscious of the severity and progression of CHF.	66 (37.7 %)
…funding for palliative care is not available.	64 (36.6 %)
…palliative care medicine is perceived to be in competition with cardiology/general medicine, internal medicine.	63 (36.0 %)

Most participants believed that HCPs do not possess sufficient knowledge about the content and the possibilities of PC. This was supported by the answer to the question about participants’ believe that the differences between general and special PC within the German health care setting is known by the HCPs. Most (n = 149; 85.1 %) believed that this distinction is not known.

The respondents also believed that death is still a taboo subject within the society and that patients do not like to accept that life is limited because of the disease. In addition, it was assumed that PC is put on the same level as euthanasia by the patients. It was considered probable that patients and relatives do not want PC to be initiated, rather, they would prefer everything possible done to preserve life. One participant stated: “a great problem is also that relatives are not able to let go [of the patient]” (#86)

Most participants also agreed to the statement that it is easier to continue the started therapy than to change it. In this context 49.1 % of the HCPs think that physicians perceive a change of therapy into PC as a defeat.

There also seems to be some barriers due to the organization of the health care system and attitudes of the HCPs. More than half of the participants agreed to the statement that palliative medicine addresses only patients with oncological diseases, and 63.4 % of the HCPs stated that they do not know whom to contact if a CHF patient is in need for PC. Nearly half of the respondents also thought that there is no PC service for patients with CHF. Health care providers believe that different attitudes towards the treatment of CHF within medical disciplines and between medical professions lead to a reduced referral rate of CHF patients to PC.

Some HCPs agreed that there are no sufficient financial conditions for PC. One participant stated: “The German Health Insurance Medical Service in Saxony tries to prevent registration of CHF patients into special PC services” (#522). Another statement implied that various medical disciplines’ financial interests prevent sufficient PC for patients with CHF (“It´s still about […] patient distribution” (#548)).

### Attitudes towards palliative care

As displayed in Table 6, HCPs agreed that the need of PC for patient with CHF exists, is rising and that via PC a more intensive care for CHF patients is possible, thus potentially enabling a better rest-of- life for the patient. Most agreed to the statement that a de-escalation of the therapy often makes more sense. In contrast to that, less than half of the participants thought that invasive medical heart failure treatment, e.g. cardiac assist devices, are reducing patients’ quality of life. One participant stated that “this way [cardiac assist devices] seems to be a practicable way […] both as bridge to transplant and destination therapy. Especially young patients […] have a long future. I would expect an escalation of the therapy of these patients” (#360).

**Table 6:** Attitudes towards palliative care

Please evaluate the following statements:	Agreement
Cardiology, general medicine, and internal medicine could learn from the expertise of palliative care medicine.	159 (90.9 %)
A more intensive care is possible via palliative care.	154 (88.0 %)
The quality of remaining life can be optimized under palliative care.	154 (88.0 %)
The demand for palliative care in treating patients with heart failure is rising.	145 (82.9 %)
The demand for palliative care in treating patients with heart failure exists.	138 (78.9 %)
De-escalation of therapy often makes more sense than continuing the present therapy.	106 (60.6 %)
Patient with chronic heart failure do not have the feeling of being in a palliative situation.	100 (57.1 %)
The quality of life in patients with advanced heart failure will further diminish with the implementation of invasive therapies like heart assist devices.	84 (48.0 %)
It is not easy to determine the right time to initiate palliative care due to the difficulty in estimating the disease’s progression.	83 (47.4)
Patients do not request palliative care.	76 (43.3 %)
Patients might refuse further escalation of therapy when palliative care is offered.	25 (14.3 %)
Palliative care can be completely taken over by the attending general practitioner/cardiologist/internist.	22 (12.6 %)
Complex heart failure-specific therapies can be performed even in very old patients. Therefore, palliative care is not necessary.	11 (6.3 %)
Great progress has been made in heart failure therapy. Therefore, palliative care is not necessary.	5 (2.9 %)

From a professional point of view, participants were aware that cardiologists, GPs and internal specialists benefit from the expertise of PC specialists. Consequently, most did not think, that non-PC specialist are able to care for the CHF patients on their own. Participants’ comments reflect the different opinions HCP have regarding who should perform PC. They ranged from “an interdisciplinary team around a patient with CHF is in the very interest of the patient” (#591) and “The integration of PC physicians at an early stage (…) is able to enhance the quality and the duration of life” (#287) to “In most cases PC could also be taken over by the general practitioner” (#197).

Slightly less than half of the participants held the opinion that PC wouldn’t be initiated because the progress of the disease is difficult to estimate or because the patient doesn’t ask for PC. One participant stated that “patients would rather ask for PC after appropriate education, often, however, there is a lack of appropriate advisory skills in the therapeutic team” (#128).

### Time to initiate palliative care

**Fig. 1:**
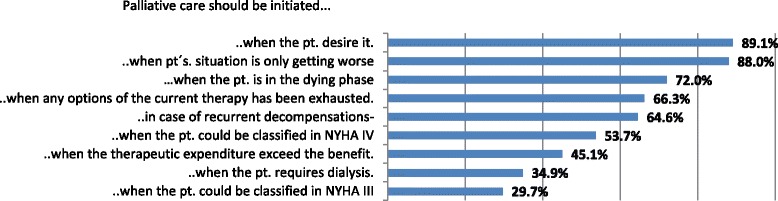
Starting Point of Palliative Care

As displayed in Fig. 1, the majority of participants agreed that PC should be initiated when patients desire it. Comments were: “If possible, the patient’s own wishes should be taken into consideration” (#190), “One should start [PC] after consulting with the patient. If the patient expresses having a high psychological strain and sends a signal that he can’t stand it any longer, [then PC should be considered]” (#68). However, some HCP stated that not the patient on his/her own should determine when to start PC. One participant commented that “it has to be considered individually, multiple criteria have to be fulfilled. Solely because the patient desires palliative care does not mean that he/she needs it” (#637). Another one stated “There should be qualified PC physicians, who appraise the present situation of the heart failure therapy before the patient is given the choice of a palliative therapeutic approach” (#360).

Most participants think that PC should start if the patient’s overall situation does not improve anymore but rather declines. Further reasons for starting PC were: if patients are facing an end-of-life situation and when all possible therapy options have been utilized. However, some participants question whether the patient benefits from PC at the end of life (“If the patient already is in the dying phase, one should evaluate if it is still useful” (#88); “If the patient is in the dying phase, he/she benefits from PC only by explaining the actual situation to the relatives and he/she is able to die in peace and the family is prepared […]” (#52)).

More than half of the participants stated that PC should start if patients consistently decompensate or could be classified in NYHA IV.

In general, most participants (n = 164; 83.4 %) thought that PC of CHF patients should be initiated earlier than it is actually practiced. One HCP states that “as already said, PC starts when a lethal disease has been diagnosed. That means, it begins with a conversation, in which the opportunity [of PC] is brought up” (#86).

Overall, comments revealed that participants regarded the optimal timing of starting PC in CHF patients as very difficult: “It’s difficult to determine the right time […]” (#591), and “This question is difficult [to answer]” (#95).

## Discussion

This is the first study evaluating barriers and facilitators to PC of patients with heart failure in Germany. Our study provides insight into physicians’ and nurses’ experiences including attitudes towards this critical issue. In summary, HCPs agree that PC for patients with CHF is needed. However, main barriers seem to be a lack of clear responsibilities, undefined communication structures, and HCP-patient-relative knowledge deficit regarding the content and the meaning of PC. In addition, HCPs shared the view that it is difficult to determine the right point of time to start PC treatment.

According to the definition of the World Health Organization [23], PC should be applied early in the course of illness. Following this advice, several conditions should be fulfilled. Firstly, heart failure patients should be educated about the course and the consequences of their disease as early as possible (e.g. at the time of diagnosis) in order to enable them to communicate theis wishes and treatment preferences with HCPs and to discuss end-of-life issues with their relatives. Secondly, patients, relatives and HCP should be informed about the content and possibilities of palliative care.

In our study HCP believe that patients and their relatives are insufficiently informed about the disease, which is supported by the result of numerous studies evaluating patients’ and relatives’ knowledge about CHF [40–42]. Evidence suggests that education is able to improve this lack of knowledge [43, 44], and should be initiated as early as possible so that patients and relatives are able to fully understand the diagnosis and its consequences. The question of “who” is responsible for patient education should be considered in light of the current structure and resources.

Another finding of this study is that HCPs in Germany consider PC as an end-of-life approach, and that they also believe that patients consider PC the same way, which is in line with results from international studies [30, 32, 45]. Health care providers, especially physicians express also their feelings about PC in terms of losing the patients or experiencing a defeat when the patients die. It seems that HCP’s attitude towards PC are based on “an inappropriate notion of ideal medicine”, in which death is something, which should not take place [46].

It is suggested to educate HCPs about PC services and aims [33], and that HCPs caring for patients with CHF should collaborate with those experienced in PC [31]. In addition, it seems important to inform the public about aims and methods of PC beyond end-of-life care, and that PC is rather a strategy to enhance quality of life. Even though evidence shows a good public understanding of PC in other countries, the public is still in need for further education [47, 48].

In our study most participants would initiate PC rather in a late stage of CHF’s course, which also has been described in another study [49]. This could be traced back towards the lack of knowledge about PC and/or CHF. Therefore, some researchers tried to develop instruments to identify patients with a PC need in the early stage of disease or to foster a PC approach [50, 51]. The use of such instruments, however, is not very common and is still under-researched. A very common approach in identifying patients in need of PC is the so called surprise question (Would I be surprised if this patient was to die in the next 6–12 months?). This questions has a high accuracy of survival prognosis in cancer patients [52] but some authors have concerns regarding the use of this questions for patients with COPD or CHF. They pointed out that this question narrows PC as end-of-life care [53, 54].

Even though in Germany PC services are at an advanced level, a clear agreement or idea of who is responsible for CHF patients does not seem to exist. There seems to be a lack of communication between the different HCPs, resulting in an insufficient care process of CHF patients. This is also reported by many researchers worldwide [30, 31, 34] and seems to be one of the main barriers to PC when treating CHF patients. To overcome this specific barrier several recommendations were made: e.g., interdisciplinary and multi-professional education targeting this theme ( PC) [33, 55–57], establishing multi-professional teams [58, 59], key workers [31], along with setting up solid pathways of care between cardiology and PC teams [56].

Professional PC can only be sufficient if the payment is sufficient for professional HCPs. Some statements made by survey participants imply that there is insufficient remuneration for PC in Germany. This barrier was identified by the legislative authority and a new law was passed in November 2015 to improve palliative and hospice care [60]. This law supports PC by defining PC as part of standard care, with appropriate remuneration and by improving the financial situation of inpatient and outpatient PC services. In addition, persons with a statutory health insurance are eligible to receive a counseling-session encompassing selection and claiming of PC services. There is hope that this will improve PC services in Germany. Our results serve as a basis for a Delphi-study to develop recommendations on how to overcome these barriers within the German health care system.

## Limitations

This study has several limitations which should be considered. For the purpose of this study we used a convenience sample of health care providers. The participants were recruited through different approaches e.g. social media, personal emails, and call for participation in professional journals. It is possible that only professionals with special interest in heart failure care or palliative care respond to our call. Furthermore, only 48.9 % of all people visiting the website containing our questionnaire did start the survey. We have no evidence-based explanation for this low rate of completed questionnaire, however, one explanation might be, that the questionnaire contained too many questions for those involved in everyday caring for this vulnerable patients. Furthermore, more than half of the participants who completed the questionnaire were male, which is a higher percentage rate of males than those working in the German health care system. But as more than half of respondents were physicians, a profession dominated by males in Germany, gender rates seem to be appropriate in relation to the underlying population. Still, generalization of results is not indicated.

In addition, we developed the questions out of our previous qualitative study. Therefore it might occur that we did not cover full range of themes regarding the topic investigated as the interviewed sample was small and local.

## Conclusion

Our findings provide insight into the attitudes of German HCPs toward and their experiences with PC. Results of our survey are mostly in agreement with those of other studies and add substantial evidence to this issue. Barriers to PC for CHF patients are mainly due to the following: lack of communication, knowledge deficit encompassing all parties (HCP, patient and relatives) regarding PC and its services of all persons involved (HCPs, patients, relatives), as well as a suspected patient knowledge deficit regarding their disease, difficulties in identifying the right time to initiate PC in CHF patients, and insufficient organizational conditions.

We could also add new findings for the discussion about barriers of PC for CHF patients. It seems that HCP lacks a sufficient commitment for PC due to their feelings that PC is not appropriate remunerated and that PC is perceived as a defeat or as a violation of the notion of ideal medicine. Furthermore, it turned out that the structure of PC in Germany is not sufficiently known even though it exists for years.

## Abbreviations

CHF, Chronic Heart Failure; COPD, Chronic obstructive pulmonary disease; GP, General practitioner; HCP, Health Care Provider; NYHA, New York Heart Association; PC, Palliative Care; SD, Standard deviation

## Additional file

Additional file 1: Questionnaire used for this study. (DOCX 40 kb)

## References

[1] Ohlmeier C, Mikolajczyk R, Frick J, Prutz F, Haverkamp W, Garbe E (2015). Incidence, prevalence and 1-year all-cause mortality of heart failure in Germany: a study based on electronic healthcare data of more than six million persons. Clin Res Cardiol.

[2] Statistisches Bundesamt. Die 10 häufigsten Todesursachen insgesamt 2016 [13.06.2016]. Available from: https://www.destatis.de/DE/ZahlenFakten/GesellschaftStaat/Gesundheit/Todesursachen/Tabellen/SterbefaelleInsgesamt.html.

[3] Ekman I, Granger B, Swedberg K, Stenlund H, Boman K (2011). Measuring shortness of breath in heart failure (SOB-HF): development and validation of a new dyspnoea assessment tool. Eur J Heart Fail.

[4] Barnes S, Gott M, Payne S, Parker C, Seamark D, Gariballa S (2006). Prevalence of symptoms in a community-based sample of heart failure patients. J Pain Symptom Manage.

[5] Leto L, Feola M (2014). Cognitive impairment in heart failure patients. J Geriatr Cardiol..

[6] Conley S, Feder S, Redeker NS (2015). The relationship between pain, fatigue, depression and functional performance in stable heart failure. Heart Lung.

[7] Corsonello A, Pedone C, Carosella L, Corica F, Mazzei B, Incalzi RA (2005). Health status in older hospitalized patients with cancer or non-neoplastic chronic diseases. BMC Geriatr.

[8] Incalzi RA, Corsonello A, Pedone C, Corica F, Carbonin P, Bernabei R (2005). Construct validity of activities of daily living scale: a clue to distinguish the disabling effects of COPD and congestive heart failure. Chest.

[9] Janssen DJ, Franssen FM, Wouters EF, Schols JM, Spruit MA (2011). Impaired health status and care dependency in patients with advanced COPD or chronic heart failure. Qual Life Res Int J Qual Life Asp Treat Care Rehab.

[10] Köberich S, Lohrmann C, Dassen T (2014). Care dependency in patients with chronic obstructive pulmonary disease and heart failure - a secondary data analysis of German prevalence studies. Scand J Caring Sci.

[11] Imes CC, Dougherty CM, Pyper G, Sullivan MD (2011). Descriptive study of partners’ experiences of living with severe heart failure. Heart Lung.

[12] Pressler SJ, Gradus-Pizlo I, Chubinski SD, Smith G, Wheeler S, Sloan R (2013). Family caregivers of patients with heart failure: a longitudinal study. J Cardiovasc Nurs.

[13] Brostrom A, Stromberg A, Dahlstrom U, Fridlund B (2003). Congestive heart failure, spouses’ support and the couple’s sleep situation: a critical incident technique analysis. J Clin Nurs.

[14] Luttik ML, Lesman-Leegte I, Jaarsma T (2009). Quality of life and depressive symptoms in heart failure patients and their partners: the impact of role and gender. J Card Fail.

[15] Martensson J, Dracup K, Canary C, Fridlund B (2003). Living with heart failure: depression and quality of life in patients and spouses. J Heart Lung Transplant..

[16] Pihl E, Jacobsson A, Fridlund B, Stromberg A, Martensson J (2005). Depression and health-related quality of life in elderly patients suffering from heart failure and their spouses: a comparative study. Eur J Heart Fail.

[17] Bekelman DB, Rumsfeld JS, Havranek EP, Yamashita TE, Hutt E, Gottlieb SH (2009). Symptom burden, depression, and spiritual well-being: a comparison of heart failure and advanced cancer patients. J Gen Intern Med.

[18] O’Leary N, Murphy NF, O’Loughlin C, Tiernan E, McDonald K (2009). A comparative study of the palliative care needs of heart failure and cancer patients. Eur J Heart Fail.

[19] Pantilat SZ, O’Riordan DL, Dibble SL, Landefeld CS (2012). Longitudinal assessment of symptom severity among hospitalized elders diagnosed with cancer, heart failure, and chronic obstructive pulmonary disease. J Hosp Med.

[20] Goodlin SJ, Kutner JS, Connor SR, Ryndes T, Houser J, Hauptman PJ (2005). Hospice care for heart failure patients. J Pain Symptom Manage.

[21] Hupcey JE (2012). The state of palliative care and heart failure. Heart Lung.

[22] Pantilat SZ, Steimle AE (2004). Palliative care for patients with heart failure. JAMA.

[23] World Health Organization. WHO Definition of Palliative Care 2015 [02.12.2015]. Available from: http://www.who.int/cancer/palliative/definition/en/.

[24] Schwarz ER, Baraghoush A, Morrissey RP, Shah AB, Shinde AM, Phan A (2012). Pilot study of palliative care consultation in patients with advanced heart failure referred for cardiac transplantation. J Palliat Med.

[25] Brannstrom M, Boman K (2014). Effects of person-centred and integrated chronic heart failure and palliative home care. PREFER: a randomized controlled study. Eur J Heart Fail.

[26] Evangelista LS, Liao S, Motie M, De Michelis N, Lombardo D (2014). On-going palliative care enhances perceived control and patient activation and reduces symptom distress in patients with symptomatic heart failure: a pilot study. Eur J Cardiovasc Nurs..

[27] Evangelista LS, Lombardo D, Malik S, Ballard-Hernandez J, Motie M, Liao S (2012). Examining the effects of an outpatient palliative care consultation on symptom burden, depression, and quality of life in patients with symptomatic heart failure. J Card Fail.

[28] Wong RC, Tan PT, Seow YH, Aziz S, Oo N, Seow SC (2013). Home-based advance care programme is effective in reducing hospitalisations of advanced heart failure patients: a clinical and healthcare cost study. Ann Acad Med Singapore.

[29] Sidebottom AC, Jorgenson A, Richards H, Kirven J, Sillah A (2015). Inpatient palliative care for patients with acute heart failure: outcomes from a randomized trial. J Palliat Med.

[30] Beernaert K, Deliens L, De Vleminck A, Devroey D, Pardon K, Van den Block L (2014). Early identification of palliative care needs by family physicians: a qualitative study of barriers and facilitators from the perspective of family physicians, community nurses, and patients. Palliat Med.

[31] Hanratty B, Hibbert D, Mair F, May C, Ward C, Capewell S (2002). Doctors' perceptions of palliative care for heart failure: focus group study. BMJ.

[32] Hupcey JE, Penrod J, Fogg J (2009). Heart failure and palliative care: implications in practice. J Palliat Med.

[33] Kavalieratos D, Mitchell EM, Carey TS, Dev S, Biddle AK, Reeve BB (2014). “Not the ‘grim reaper service”: an assessment of provider knowledge, attitudes, and perceptions regarding palliative care referral barriers in heart failure. J Am Heart Assoc.

[34] Browne S, Macdonald S, May CR, Macleod U, Mair FS (2014). Patient, carer and professional perspectives on barriers and facilitators to quality care in advanced heart failure. PLoS One.

[35] World Health Organization. Global Atlas of Palliative Care at the End of Life 2014. Available from: http://www.who.int/nmh/Global_Atlas_of_Palliative_Care.pdf.

[36] HOPE 2015. HOPE 2015 Tabellen Basisbogen. 2015 [13.06.2016]. Available from: https://www.hope-clara.de/download/2015_HOPE_Basistabellen.pdf.

[37] Ziehm J, Farin-Glattacker E, Seibel K, Becker G, Köberich S. Healthcare Professionals' Attitudes Regarding Palliative Care for Patients with Chronic Heart Failure: An Interview Study BMC Palliative Care. submitted. 10.1186/s12904-016-0149-9 PMC4986383 27526940

[38] Mayring P (2010). Qualitative Inhaltsanalyse: Grundlagen und Techniken.

[39] Deutsche Gesellschaft für Palliativmedizin. Definition of general ambulant palliative care [Definition der Allgemeinen ambulanten Paliativversorgung] 2009 [13.06.2016]. Available from: https://www.dgpalliativmedizin.de/allgemein/allgemeine-ambulante-palliativversorgung-aapv.html.

[40] Dracup K, Moser DK, Pelter MM, Nesbitt T, Southard J, Paul SM (2014). Rural patients’ knowledge about heart failure. J Cardiovasc Nurs.

[41] Rodriguez KL, Appelt CJ, Switzer GE, Sonel AF, Arnold RM (2008). “They diagnosed bad heart”: a qualitative exploration of patients’ knowledge about and experiences with heart failure. Heart Lung.

[42] Klindtworth K, Oster P, Hager K, Krause O, Bleidorn J, Schneider N (2015). Living with and dying from advanced heart failure: understanding the needs of older patients at the end of life. BMC Geriatr.

[43] Boyde M, Song S, Peters R, Turner C, Thompson DR, Stewart S (2013). Pilot testing of a self-care education intervention for patients with heart failure. Eur J Cardiovasc Nurs..

[44] Roncalli J, Perez L, Pathak A, Spinazze L, Mazon S, Lairez O (2009). Improvement of young and elderly patient’s knowledge of heart failure after an educational session. Clin Med Cardiology.

[45] Swetz KM, Shanafelt TD, Drozdowicz LB, Sloan JA, Novotny PJ, Durst LA (2012). Symptom burden, quality of life, and attitudes toward palliative care in patients with pulmonary arterial hypertension: results from a cross-sectional patient survey. J Heart Lung Transplant..

[46] Momeyer R (1995). Does physician assisted suicide violate the integrity of medicine?. J Med Philos.

[47] MacLeod RD, Thompson R, Fisher JW, Mayo K, Newman NW, Wilson DM (2012). New Zealanders’ knowledge of palliative care and hospice services. N Z Med J.

[48] McIlfatrick S, Noble H, McCorry NK, Roulston A, Hasson F, McLaughlin D (2014). Exploring public awareness and perceptions of palliative care: a qualitative study. Palliat Med.

[49] Gelfman LP, Kalman J, Goldstein NE (2014). Engaging heart failure clinicians to increase palliative care referrals: overcoming barriers, improving techniques. J Palliat Med.

[50] Strachan PH, Joy C, Costigan J, Carter N (2014). Development of a practice tool for community-based nurses: the Heart Failure Palliative Approach to Care (HeFPAC). Eur J Cardiovasc Nurs..

[51] Thoonsen B, Engels Y, van Rijswijk E, Verhagen S, van Weel C, Groot M (2012). Early identification of palliative care patients in general practice: development of RADboud indicators for PAlliative Care Needs (RADPAC). Br J Gen Pract..

[52] Moroni M, Zocchi D, Bolognesi D, Abernethy A, Rondelli R, Savorani G (2014). The ‘surprise’ question in advanced cancer patients: A prospective study among general practitioners. Palliat Med.

[53] Murray S, Boyd K (2011). Using the ‘surprise question’ can identify people with advanced heart failure and COPD who would benefit from a palliative care approach. Palliat Med.

[54] Small N, Gardiner C, Barnes S, Gott M, Payne S, Seamark D (2010). Using a prediction of death in the next 12 months as a prompt for referral to palliative care acts to the detriment of patients with heart failure and chronic obstructive pulmonary disease. Palliat Med.

[55] Afshar K, Geiger K, Muller-Mundt G, Bleidorn J, Schneider N (2015). [Generalist palliative care for non-cancer patients : a review article]. Schmerz (Berlin, Germany).

[56] Johnson MJ, Maccallum A, Butler J, Rogers A, Sam E, Fuller A (2012). Heart failure specialist nurses’ use of palliative care services: a comparison of surveys across England in 2005 and 2010. Eur J Cardiovasc Nurs..

[57] Kirolos I, Tamariz L, Schultz EA, Diaz Y, Wood BA, Palacio A (2014). Interventions to improve hospice and palliative care referral: a systematic review. J Palliat Med.

[58] Davidson PM, Paull G, Introna K, Cockburn J, Davis JM, Rees D (2004). Integrated, collaborative palliative care in heart failure: the St. George heart failure service experience 1999-2002. J Cardiovasc Nurs.

[59] Lindvall C, Hultman TD, Jackson VA (2014). Overcoming the barriers to palliative care referral for patients with advanced heart failure. J Am Heart Assoc.

[60] Bundesministerium für Gesundheit. Bundestag beschließt Gesetz zur Verbesserung der Hospiz- und Palliativversorgung 2015 [17.12.2015]. Available from: http://www.bmg.bund.de/ministerium/meldungen/2015/hpg-bt-23-lesung.html.

