# Supplementary material for: Palliative care for patients with heart failure: facilitators and barriers - a cross sectional survey of German health care professionals
Source: BMC Health Serv Res. 2016 Aug 8;16:361. doi: 10.1186/s12913-016-1609-x (PMC4977661; doi:10.1186/s12913-016-1609-x)
Supplement: Additional file 1: — Questionnaire used for this study. (DOCX 40 kb) [file 12913_2016_1609_MOESM1_ESM.docx]

Supplementary file 1: Questionnaire used for this study

| **A** | **We would like to know how you personally define “Palliative care” and what you expect from “Palliative care”**  **To what extent do you agree to the following statements?**  **Within palliative care…** | | | | | |
| --- | --- | --- | --- | --- | --- | --- |
|  |  | **I fully agree** | **I somewhat agree** | **Neither nor** | **I rather disagree** | **I do not agree at all** |
| A1 | ……an incurable patient will be cared for. |  |  |  |  |  |
| A2 | … physical symptoms and discomfort as well as their relief are paramount. |  |  |  |  |  |
| A3 | … the aim is to achieve/maintain best possible quality of life. |  |  |  |  |  |
| A4 | … one tries to avoid unnecessary therapy. |  |  |  |  |  |
| A5 | … psychological support is provided. |  |  |  |  |  |
| A6 | … spiritual support will be provided. |  |  |  |  |  |
| A7 | … the aim is a dignified death. |  |  |  |  |  |
| A8 | … extent and intensity of technical and life-sustaining measures well be deliberated and discussed with the patient. |  |  |  |  |  |
| A9 | … communication with and involvement of the relatives plays an important role. |  |  |  |  |  |

If you would like to make further comments about palliative care, please state here.

| **B** | **We would like to know your opinion about which organizational conditions have to be in place to carry out palliative care for patients with chronic heart failure.**  **To what extent do you agree to the following statements?**  **In favor of palliative care for patients with chronic heart failure….** | | | | | |
| --- | --- | --- | --- | --- | --- | --- |
|  |  | **I fully agree** | **I somewhat agree** | **Neither nor** | **I rather disagree** | **I do not agree at all** |
| B1 | …cardiologist, general practitioner, palliative care practitioner and internist as well as nurses should work cooperatively together (meetings, case conferences, etc.). |  |  |  |  |  |
| B2 | …palliative care practitioner should have only an advisory role. |  |  |  |  |  |
| B3 | …clear arrangements between all professionals/disciplines should be made. |  |  |  |  |  |
| B4 | …palliative care practitioner should be available for consultation. |  |  |  |  |  |
| B5 | …palliative care should be initiated by the attending palliative care practitioner. |  |  |  |  |  |
| B6 | …palliative care should be initiated by the attending cardiologist. |  |  |  |  |  |
| B7 | …palliative care should be initiated by the attending general practitioner. |  |  |  |  |  |
| B8 | …palliative care should be initiated by the attending internist. |  |  |  |  |  |
| B9 | …palliative care should be initiated by a nurse. |  |  |  |  |  |
| B10 | …further education in the area of palliative care should be offered to all professions. |  |  |  |  |  |
| B11 | …collective interdisciplinary education should be offered to all physicians involved in caring for the patient. |  |  |  |  |  |
| B12 | …palliative care should be established within the institution (hospitals/long-term care facilities). |  |  |  |  |  |
| B13 | ..therapy should mainly be carried out by the palliative care practitioner. |  |  |  |  |  |
| B14 | ..therapy should mainly be carried out by the attending cardiologist. |  |  |  |  |  |
| B15 | ..therapy should mainly be carried out by the attending general practitioner. |  |  |  |  |  |
| B16 | ..therapy should mainly be carry out by the attending internist. |  |  |  |  |  |

If you would like to make further comments about organizational conditions to carry out palliative care for patient with chronic heart failure, please state here.

| **C** | **We would like to know which barriers do you perceive regarding palliative care of patients with chronic heart failure**  **To what extent do you agree to the following statements?**  **Palliative care of patients with chronic heart failure is often hampered because…** | | | | | |
| --- | --- | --- | --- | --- | --- | --- |
|  |  | **I fully agree** | **I somewhat agree** | **Neither nor** | **I rather disagree** | **I do not agree at all** |
| C1 | …patients and relatives are not sufficiently informed about the severity and the prognosis of CHF. |  |  |  |  |  |
| C2 | …physicians and nurses have an information deficit about content and possibilities of palliative care. |  |  |  |  |  |
| C3 | …physicians/nurses do not have a palliative care contact person when needed. |  |  |  |  |  |
| C4 | …no palliative care approach exists for patients with heart failure. |  |  |  |  |  |
| C5 | …palliative care medicine mainly focuses on oncological patients. |  |  |  |  |  |
| C6 | …patients put palliative care medicine on the same level as euthanasia. |  |  |  |  |  |
| C7 | …patients have a degree of reluctance in accepting that life is limited. |  |  |  |  |  |
| C8 | ...the creeping course of the disease does not look threatening.. |  |  |  |  |  |
| C9 | …the medical team is not conscious of the severity and progression of CHF. |  |  |  |  |  |
| C10 | … a lot of physicians perceive palliative care as a defeat. |  |  |  |  |  |
| C11 | …in our society dying is a taboo subject. |  |  |  |  |  |
| C12 | …it is easier to continue with an existing therapy than to discuss a change of the therapy´s aim with the patient. |  |  |  |  |  |
| C13 | …palliative care medicine is perceived to be in competition with cardiology/general medicine, internal medicine. |  |  |  |  |  |
| C14 | ...there are different attitudes between the different medical professions (nurses, physicians) regarding therapy of patients with heart failure. |  |  |  |  |  |
| C15 | ...there are different attitudes between the different medical disciplines (cardiology, general medicine, internal medicine, palliative care medicine) regarding therapy of patients with heart failure. |  |  |  |  |  |
| C16 | …patients want everything possible to be done. |  |  |  |  |  |
| C17 | …relatives want everything possible to be done. |  |  |  |  |  |
| C18 | …funding for palliative care is not available. |  |  |  |  |  |

| **D** | **We would like to know if you - in general - consider palliative care for patients with chronic heart failure as useful.**  **Mark with a cross the statement you most agree with.** |
| --- | --- |
|  | Yes, I consider palliative care of patients with chronic heart failure as useful |
|  | No, I don´t consider palliative care of patients with chronic heart failure as useful |
|  | I have no opinion about that issue |

| **D** | **Please evaluate the following statements** | | | | | |
| --- | --- | --- | --- | --- | --- | --- |
|  |  | **I fully agree** | **I somewhat agree** | **Neither nor** | **I rather disagree** | **I do not agree at all** |
| D1 | The demand for palliative care in treating patients with heart failure exists. |  |  |  |  |  |
| D2 | The demand for palliative care in treating patients with heart failure is rising. |  |  |  |  |  |
| D3 | De-escalation of therapy often makes more sense than continuing the present therapy. |  |  |  |  |  |
| D4 | Cardiology, general medicine, and internal medicine could learn from the expertise of palliative care medicine. |  |  |  |  |  |
| D5 | A more intensive care is possible via palliative care. |  |  |  |  |  |
| D6 | The quality of life in patients with advanced heart failure will further diminish with the implementation of invasive therapies like heart assist devices. |  |  |  |  |  |
| D7 | The quality of remaining life can be optimized under palliative care. |  |  |  |  |  |
| D8 | Patients do not request palliative care. |  |  |  |  |  |
| D9 | It is not easy to determine the right time to initiate palliative care due to the difficulty in estimating the disease’s progression. |  |  |  |  |  |
| D10 | Complex heart failure-specific therapies can be performed even in very old patients. Therefore, palliative care is not necessary. |  |  |  |  |  |
| D11 | Great progress has been made in heart failure therapy. Therefore, palliative care is not necessary. |  |  |  |  |  |
| D12 | Palliative care can be completely taken over by the attending general practitioner/cardiologist/internist. |  |  |  |  |  |
| D13 | Patient with chronic heart failure do not have the feeling of being in a palliative situation. |  |  |  |  |  |
| D14 | Patients might refuse further escalation of therapy when palliative care is offered. |  |  |  |  |  |

If you have any further reasons for or against palliative care of patients with chronic heart failure, please state here

| **E** | **We would like to know your opinion about the right time to start palliative care of patients with chronic heart failure**  **To what extent do you agree to the following statements?**  **Palliative care of patients with chronic heart failure should start…** | | | | | |
| --- | --- | --- | --- | --- | --- | --- |
|  |  | **I fully agree** | **I somewhat agree** | **Neither nor** | **I rather disagree** | **I do not agree at all** |
| E1 | …when any options of the current therapy has been exhausted. |  |  |  |  |  |
| E2 | …when the patient desire it. |  |  |  |  |  |
| E3 | …when the patients could be classified in NYHA III. |  |  |  |  |  |
| E4 | … when the patients could be classified in NYHA IV. |  |  |  |  |  |
| E5 | …when the patients requires dialysis. |  |  |  |  |  |
| E6 | …when the therapeutic expenditure exceed the benefit. |  |  |  |  |  |
| E7 | …in case of recurrent decompensations. |  |  |  |  |  |
| E8 | …when patient´s situation is only getting worse. |  |  |  |  |  |
| E9 | …when the patients is in the dying phase. |  |  |  |  |  |
| E10 | Generally palliative care should start earlier than it used to be. |  |  |  |  |  |

If you have further remarks regarding the time to start palliative of patients with chronic heart failure, please state here.

| **F** | **Now, we would like to know, if you think that the differences between general palliative care and specialized palliative care is known within the health care system**  **Mark with a cross the statement you most agree with.** |
| --- | --- |
|  | Yes, I think that the differences between general palliative care and specialized palliative care is known within the health care system |
|  | No, I don´t think that the differences between general palliative care and specialized palliative care is known within the health care system |
